# High Neutrophil–Lymphocyte Ratio and Low Lymphocyte–Monocyte Ratio Combination after Thrombolysis Is a Potential Predictor of Poor Functional Outcome of Acute Ischemic Stroke

**DOI:** 10.3390/jpm12081221

**Published:** 2022-07-27

**Authors:** Farzaneh Sadeghi, Ferenc Sarkady, Katalin S. Zsóri, István Szegedi, Rita Orbán-Kálmándi, Edina G. Székely, Nikolett Vasas, Ervin Berényi, László Csiba, Zsuzsa Bagoly, Amir H. Shemirani

**Affiliations:** 1 Division of Clinical Laboratory Sciences, Department of Laboratory Medicine, Faculty of Medicine, University of Debrecen, 4032 Debrecen, Hungary; farzanehsadeghi92@yahoo.com (F.S.); sarkadyf@gmail.com (F.S.); kalmandi.rita@med.unideb.hu (R.O.-K.); egszekely@gmail.com (E.G.S.); bagoly@med.unideb.hu (Z.B.); 2 Central Pharmacy, Szent Borbála Hospital, 2800 Tatabánya, Hungary; zskatika@gmail.com; 3 Doctoral School of Neuroscience, Department of Neurology, Faculty of Medicine, University of Debrecen, 4032 Debrecen, Hungary; iszegedi@med.unideb.hu (I.S.); vasasn@gmail.com (N.V.); 4 Department of Radiology, Faculty of Medicine, University of Debrecen, 4032 Debrecen, Hungary; eberenyi@med.unideb.hu (E.B.); csiba@med.unideb.hu (L.C.); 5 ELKH-DE Cerebrovascular and Neurodegenerative Research Group, 4032 Debrecen, Hungary; 6 Central Laboratory, Szent Borbála Hospital, 2800 Tatabanya, Hungary

**Keywords:** neutrophil-lymphocyte ratio, lymphocyte-monocyte ratio, ischemic stroke, thrombolysis

## Abstract

(1) Background: Ischemic stroke is one of the leading causes of death and disability. An inflammatory response is observed in multiple stages of cerebral ischemia, particularly in the acute phase. Recent publications revealed that the neutrophil–lymphocyte ratio (NLR) and lymphocyte–monocyte ratio (LMR) may be used to predict long-term prognosis in acute ischemic stroke (AIS) after thrombolysis. To test whether there is a relationship between the combination of these parameters and long-term prognosis, we analyzed the NLR–LMR combination in AIS patients treated with intravenous recombinant tissue plasminogen activator (rtPA); (2) Methods: The study included 285 adults with a diagnosis of AIS and rtPA treatment within a 4.5 h time window. Blood samples were obtained at admission and 24 h after thrombolysis to calculate pre- and post-thrombolysis NLR and LMR. Clinical data, including NIHSS was registered on admission and day 1. The long-term outcome was defined 90 days post-event by the modified Rankin Scale (mRS). Therapy-associated intracranial hemorrhage (ICH) was classified according to ECASS II. Receiver operating characteristic curve (ROC) analysis was performed to determine optimal cutoffs of NLR and LMR as predictors of therapy outcomes; (3) Results: Patients were stratified by cutoffs of 5.73 for NLR and 2.08 for LMR. The multivariate logistic regression model, including all possible confounders, displayed no significant association between NLR or LMR with 3-months functional prognosis. The combination of high NLR–low LMR vs. low NRL–high LMR as obtained 24 h after thrombolysis was found to be an independent predictor of poor 3-months functional outcome (mRS ≥ 2; OR 3.407, 95% CI 1.449 to 8.011, *p* = 0.005). The proportion of patients between low NLR–high LMR and high NLR–low LMR groups from admission to day 1 showed no significant change in the good outcome group. On the other hand, in the poor outcome group (mRS ≥ 2), low NLR–high LMR and high NLR–low LMR groups displayed a significant shift in patient proportions from 67% and 21% at admission (*p* = 0.001) to 36% and 49% at 24 h after thrombolysis (*p* < 0.001), respectively; (4) Conclusions: Our study demonstrated for the first time that a high NLR–low LMR combination as observed at 24 h after thrombolysis can serve as an independent predictor of 3-months poor outcome in AIS patients. This simple and readily available data may help clinicians to improve the prognostic estimation of patients and may provide guidance in selecting patients for personalized and intensified care post-thrombolysis.

## 1. Introduction

Despite recent advances in the treatment of acute ischemic stroke (AIS), the disease puts a heavy burden on individuals, families, and the health care system. Stroke is ranked the second most common cause of death and the third leading cause of death and disability in the world. Globally, in 2019, there were 6.5 million deaths from stroke and 143 million disability-adjusted life-years due to stroke [1]. Although mechanical thrombectomy has revolutionized stroke care in the past decade [2], intravenous (i.v.) thrombolysis with recombinant tissue plasminogen activator (rt-PA) remains the most commonly used pharmacological therapy for AIS. Recent data has shown that treatment within the first hour of symptom onset produces significantly improved rates of morbidity and mortality [3]. This remarkable 60 min period from the onset of symptoms is often referred to as The Golden Hour. rt-TPA is the current gold standard for the treatment of AIS [4]. With the extended time-window of 4.5 h [5], approximately half of the patients receiving thrombolysis attain total or nearly total neurological recovery at 3 months [6]. On the other hand, a large proportion of patients will not benefit from thrombolysis, moreover, 6–8% of treated patients will develop potentially fatal intracerebral hemorrhage. The identification of patients in whom thrombolysis will not be effective or would cause potential side-effects could be a key approach to personalize acute stroke care, improve long-term quality of life, and reduce the global burden of AIS. To achieve this, a rapid, cheap, simple, easily accessible, and reliable prognostic marker of thrombolysis outcome is needed. Neutrophil–lymphocyte ratio (NLR) and lymphocyte–monocyte ratio (LMR) have been shown to be associated with various pathological conditions [7,8,9]. NLR and LMR are potential novel biomarkers of inflammation and immune response [10]. They simply drive from complete blood count, and, in this way, they are rapidly and readily available markers for clinical use before administering the thrombolytic agent. Recently, traces of evidence indicated a causal link between the prognosis of AIS, NLR, and LMR levels [11,12]. Here, we analyzed the potential relationship between NLR and LMR levels and the 3-months outcome of AIS after intravenous rtPA administration.

## 2. Methods

### 2.1. Study Population

Enrolled in this study were consecutive AIS patients admitted to the Department of Neurology, the University of Debrecen, Hungary, between September 2016 and April 2018. Inclusion and exclusion criteria of patients were identical to the standard criteria of intravenous rtPA administration according to the 2008 ESO guideline [13]. The diagnosis of ischemic stroke was confirmed by using non-contrast computerized tomography (NCCT) scan and computed tomography angiography (CTA). Clinical data, including the National Institutes of Health Stroke Scale (NIHSS), was registered on admission and day 1. Thrombolysis was performed within the 4.5 h time window from the onset of symptoms using intravenous rtPA according to standard protocols [13]. Patients receiving mechanical thrombectomy in addition to thrombolysis were not included in the study. A control NCCT was performed on day 1 and early ischemic changes based on admission NCCT and day 1 NCCT were calculated using the Alberta Stroke Program Early CT Score (ASPECTS) as assessed by four independent radiologists [14]. Stroke etiology was defined by the Trial of ORG 10,172 according to the Acute Stroke Treatment (TOAST) criteria [15]. The presence of intracerebral hemorrhage (ICH) was tested on day 1 using NCCT, and patients with hemorrhage were divided into two groups: symptomatic (SICH) or asymptomatic (aSICH), based on the European Cooperative Acute Stroke Study (ECASS) II criteria [5]. A short-term outcome evaluation was performed on day 1 after thrombolysis. The favorable short-term outcome was defined as a decrease in the NIHSS score by at least 4 points or to 0, while a poor short-term outcome was defined as an increase in NIHSS score by at least 4 points [16]. The modified Rankin Scale (mRS) was registered to define long-term outcome at 90 days. Unfavorable outcome was defined as mRS greater than 1 (mRS ≥ 2). 

The study was approved by the Ethics Committee of the University of Debrecen, Hungary, and the Ethics Board of the Medical Research Council of the Hungarian Ministry of Human Capacities, Hungary. The study protocol conformed to the ethical guidelines of the 1975 Declaration of Helsinki. All patients or their relatives provided written informed consent.

### 2.2. Blood Sampling and Laboratory Measurements

Peripheral blood samples were drawn before the initiation of rt-PA infusion and 24 h after thrombolysis. From the blood samples taken on admission, routine laboratory examinations were performed (ions, glucose levels, renal and liver function tests, high-sensitivity C-reactive protein (hsCRP)) by standard methods (Roche Diagnostics, Mannheim, Germany).

Complete blood counts were assessed from blood samples obtained before and 24 h after thrombolysis using an automated analyzer (XE 2100, Sysmex Europe GmbH, Hamburg, Germany). Hematological parameters were determined immediately after blood collection. The absolute neutrophil-to-lymphocyte count (NLR), and the absolute lymphocyte-to-monocyte count (LMR) were calculated from blood samples obtained on both occasions. 

### 2.3. Statistical Methods

Continuous variables were expressed either as mean ± SD or median and interquartile range (IQR) as appropriate. Categorical data were expressed as numbers and percentages. Multiple groups of continuous data were compared using one-way analysis of variance (ANOVA) using the Bonferroni post-hoc test or Kruskal–Wallis analysis with the Dunn–Bonferroni post-hoc test. Categorical data were compared using the χ^2^ of Fisher’s exact tests where appropriate. Receiver operating characteristic (ROC) curves were built by plotting sensitivity vs. 1-specificity and calculating the area under the curve (AUC). Optimal threshold values were calculated based on Youden’s J statistics. Multivariable logistic regression models were used to test the independent effect of NLR, LMR, their combination, or each leukocyte sub-type count on outcome measures before and after adjustment for major baseline characteristics. A *p*-value less than 0.05 was considered significant. Statistical analyses were performed using SPSS 18.0 (Chicago, IL, USA) and MedCalc 14.8.1 software (Mariakerke, Belgium).

## 3. Results

### 3.1. Baseline Characteristics of Enrolled Patients

During the study period, a total of 285 consecutive AIS patients undergoing i.v. thrombolysis treatment were enrolled in the study. Baseline characteristics of included patients are listed in Table 1. The mean age of the patients was 66 ± 12.9 years, with 44.2% being female. The median baseline NIHSS score was 6 (IQR (5, 9.1)) and median 90-day mRS was 1.0. Patients with poor outcome (mRS ≥ 2) at 90 days after stroke were significantly older, had higher blood pressure and atrial fibrillation more frequently, and more severe neurological deficit on admission as compared to those with a good outcome. Moreover, patients with poor outcome showed significantly higher NLR and significantly lower LMR as compared to those with good outcome (Table 1).

### 3.2. White Blood Cell Counts, NLR and LMR during Thrombolysis

In the total cohort, the median neutrophil count, monocyte count, and NLR increased, whereas median LMR decreased 24 h after thrombolysis when compared with admission results (Table 2). An inverse but modest correlation was found between neutrophil count and lymphocyte count at admission (*r* = −0.166, *p* = 0.002) and at day 1 (*r* = −0.200, *p* = 0.001). Significant but modest positive correlation was found between lymphocyte count and monocyte count at admission (*r* = 0.261, *p* < 0.001) but not at day 1. Neutrophil count also correlated with monocyte count at admission (*r* = 0.381, *p* < 0.001) and at day 1 (*r* = 0.598, *p* < 0.001). 

Results are depicted as mean ± SD or median (interquartile range). ACE: angiotensin converting enzyme. ASPECTS: Alberta Stroke Program Early CT Score. BA: basilar artery. BMI: body mass index. DM: diabetes mellitus. hsCRP: high sensitivity C reactive protein measurement. ICA: internal carotid artery. LMR: lymphocyte–monocyte ratio. mRS: modified Rankin Scale. NIHSS: National Institutes of Health Stroke Scale. NLR: neutrophil–lymphocyte ratio. PAD: peripheral artery disease. SICH: symptomatic intracerebral hemorrhage. aSICH: asymptomatic intracerebral hemorrhage. TIA: transient ischemic attack. TOAST: Trial of ORG 10,172 in Acute Stroke Treatment. WBC: white blood cell count.

None of the leukocyte indices showed association with stroke etiology, stroke severity at admission, or with hemorrhagic transformation at admission (Table 3 and Supplementary Table S1). On the other hand, neutrophil count, monocyte count, and NLR significantly increased, while lymphocyte count and LMR significantly decreased 24 h post-rtPA in patients with more severe stroke. A similar pattern was seen in those who suffered therapy-associated intracerebral hemorrhage (Table 3).

Neutrophil count and NLR were significantly higher in the ASPECTS 10-8 group compared with the ASPECTS ≤7 group at day 1 (Supplementary Table S2). Univariate logistic regression proved a significant protective effect of higher LMR at admission with functional dependence at 3 months post-event (OR = 0.755, 95%CI (0.631, 0.903), *p* = 0.002) (Supplementary Table S3). Similar analysis showed no association between NLR and long-term functional outcome. In addition to LMR, age, hypertension, atrial fibrillation, and stroke characteristics (NIHSS, hemorrhagic transformation, and stroke localization) showed association with long-term outcome of therapy, but in the multivariate model only age, NIHSS, hemorrhagic transformation, and stroke localization remained as significant variables (Supplementary Table S3). Although in the univariate model, NLR and LMR, measured at 24 h after rtPA therapy, showed a highly significant association with functional outcomes at 3 months post-event, a multivariate logistic regression model, including all possible confounders, displayed no significant association of these parameters with 3-months functional outcome (Supplementary Table S4).

**Figure 1:**
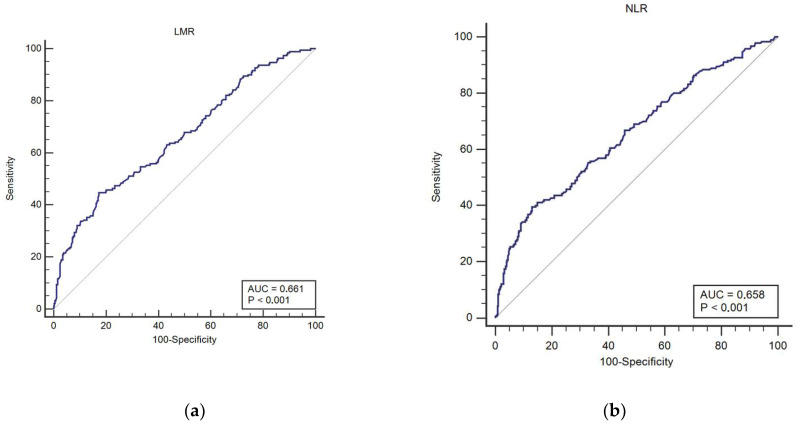
Receiver operating characteristics (ROC) curve analysis of admission neutrophil-to-lymphocyte ratio (NLR) (**a**) and lymphocyte-to-monocyte ratio (LMR) (**b**) values predicting functional dependence (mRS2) at 3 months post-event.

At baseline, the median values of NLR and LMR of the study population were 2.9 (inter-quartile range, IQR (1.94, 4.82) and 3.22 (IQR (2.42, 4.29)), respectively. The optimal threshold values for the prediction of poor functional outcome at 3 months post-event based on the best Youden index by ROC analysis were 5.73 for NLR and 2.08 for LMR (Figure 1). According to the optimal cutoff values of NLR and LMR at admission, patients were classified into four groups: low NLR–high LMR, high NLR–high LMR, low NLR–low LMR, and high NLR–low LMR. 

**Figure 2:**
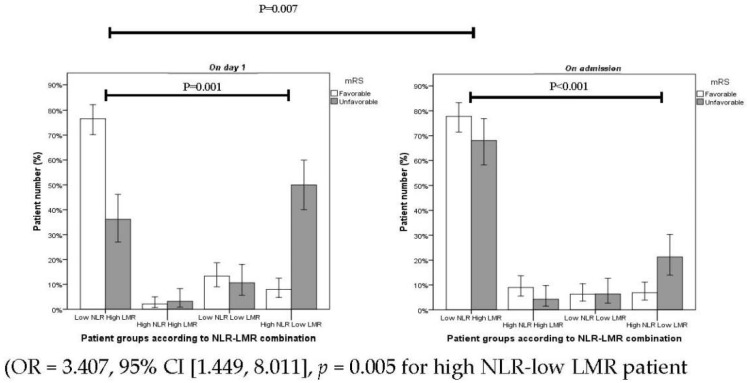
Proportion of patients in different NLR–LMR combination groups on admission and on day 1.

Out of 190 patients with favorable outcome, 77% of patients fell into the category of low NLR–high LMR combinations, while the high NLR–low LMR group contained only 6.8% of patients at admission. The proportion of patients with favorable outcome as stratified according to low NLR–high LMR and high NLR–low LMR were 76% and 7.8% at day 1, respectively. Out of 95 patients in the poor outcome group, 67% of patients were stratified as low NLR–High LMR, while only 21% were stratified as high NLR–low LMR before the administration of thrombolysis (*p* = 0.001) (Figure 2). At 24 h after thrombolysis, the proportion of patients with poor outcome displayed a significant shift in the above groups as 36% of patients could be stratified as having low NLR–high LMR while the high NLR–low LMR group included 49% (*p* < 0.001) of patients (Figure 2). 

The combination of NRL and LMR as determined at 24 h after thrombolysis was found to be an independent predictor of poor functional outcome at 3 months post-event (OR = 3.407, 95% CI [1.449, 8.011], *p* = 0.005 for high NL–low LMR patients. 

Group vs. low NRL–high LMR patient group) after controlling for all potential confounders (Table 4).

## 4. Discussion

The present study, to the best of our knowledge, is the first to provide and discuss the evidence in support of a combination of measured NLR and LMR at 24 h post-thrombolysis as an independent prognostic factor that possesses clinical significance and feasibility to identify 3-months poor outcome of AIS patients treated with intravenous thrombolysis. Confirmed by a multivariate analysis, in addition to gender, NIHSS, and TOAST classification, high NLR–low LMR remained an independent prognostic factor for 3-months poor outcome in AIS patients after rtPA.

Inflammation has a very important role in ischemic brain injury. Ischemia following stroke activates microglia, and, consequently, blood-derived immune cells infiltration into the brain tissue follows within hours to a few days [17,18]. Neutrophils reach the infarct area within the first few hours of brain infarction, monocytes during the first 24 h, and lymphocytes between 24 and 48 h [19,20,21]. Immune cells release reactive oxygen species and a variety of inflammatory mediators, adhesion molecules, cytokines, chemokines, and proteases which exacerbate tissue damage [22]. Experimental stroke models have shown increased hematopoiesis and greater output of neutrophils from the bone marrow via increased stimulation of the autonomic nervous system post-stroke [23]. Post-thrombolysis NLR as a marker of increased risk for poor outcome within 3 months after stroke onset has been also found by others [24]. Another research group analyzed the ability of admission NLR for 90-day stroke outcome prediction after endovascular stroke therapy [25]. They found NLR (cutoff point at ≥5.9) a valuable prognostic marker for poor outcome. Our results are in line with others with no independent association between infarct size and NLR or LMR after 24 h post-event [26]. Pektezel et al. conducted a retrospective evaluation of acute stroke patients treated with rtPA by comparing favorable outcome (mRS ≤ 3) with excellent outcome (mRS 0 or 1) at admission and after 24 h [27]. This study showed that significant elevation of NLR from admission to 24 h post-event, and NLR ≤ 3.6 after 24 h revealed favorable prognosis. They conclude that elevated NLR during the first 24 h is an epiphenomenon of poor prognosis. On the contrary, a recent meta-analysis could not find sufficient data to prove this point [28]. Shi et al. found no significant difference in the NRL at admission of patients with good versus poor outcome. We experienced a similar cutoff point for NLR (at ≥5.73), and NRL did not show significant difference at admission between poor and good outcome groups [24]. Others found, in a small group of patients, no significant relation between NRL or LMR and long-term outcome in AIS patients treated by thrombolysis [29]. 

Monocytes release inflammatory mediators, such as chemokines, intercellular adhesion molecule1, interleukin-1, IL-6, IL-8, and tumor necrosis factor, and contribute to the development of inflammation by cerebral ischemia and hypoxia in the area of brain infarct. Furthermore, monocytes promote thrombosis and vascular occlusion by forming platelet monocyte aggregates, which aggravate ischemic injury [30]. In the ischemic brain, the monocyte-derived macrophages (MDMs) differentiate from monocytes [31]. MDMs are potent phagocytic cells and are involved in the long-term spontaneous functional recovery of the brain after ischemia [32]. LMR at admission was also evaluated for 3-months prognosis in patients with stroke and thrombolysis therapy [33]. They demonstrated that a higher LMR value (cutoff point at 3.48) was an independent factor to predict the clinical outcome of stroke before rtPA administration. 

The exploration of temporal changes in levels of peripherally circulating leukocytes demonstrated that immediately after ischemic stroke there is an exponential decrease in the lymphocyte count [34]. Lower lymphocyte counts, as a marker of severe brain damage, were predictive of poor neurological improvement and poor functional outcome after stroke [35]. Our analysis demonstrated a similar trend in that the number of patients with poor outcome were significantly increased in the high NLR–low LMR group within one day after thrombolysis. This shows the relative association of a low lymphocyte count with poor outcome. One possible mechanism is that the number of regulatory T-cells and B-cells as disease-limiting protective cells, which maintain immune homestasis and produce anti inflammatory agents, increase in the brain tissue after ischemic injury [36]. The subset of leukocytes determines the population of adrenergic receptors and cholinergic receptors on the cell surface. A high number of cholinergic receptors and low number of adrenergic receptors express on the surface of lymphocytes, whereas granulocytes bear a high density of adrenergic receptors and a low density of cholinergic receptors [37]. Another proposed explanation is that a low lymphocyte count reflects elevated sympathetic tone and cortisol level, which can increase the production of proinflammatory cytokines, such as IL-6 and tumor necrosis factor, that aggravate ischemic injury [38]. Similarly, our results also demonstrated that a lower lymphocyte count had an obvious association with poor functional outcome after three months.

We found, as have others, that there is no correlation between aSICH, stroke severity, and leukocytes indices determined at admission [26,39,40]. The relation between leukocyte profile and stroke outcome after mechanical thrombectomy has also been investigated [26,41]. In one study, higher counts of neutrophils and NLR at admission and at day 1, as well as lower lymphocyte counts at day 1 were associated with poor prognosis (mRS > 2) [41]. In another study, higher NLR and lower LMR 24 h after mechanical thrombectomy but not at admission were significant predictors of mRS at 3-months functional outcome [26]. They found optimal cutoff values of 5.5 for NLR and 2.0 for LMR after thrombectomy. 

Our study is the first to discuss the value of the combined post-thrombolysis high NLR–low LMR ratio in evaluating the prognosis of AIS patients at 3 months post-event. Our findings are corroborated by the observations of previous studies, which had revealed the lack of reliability of the pre-thrombolysis prognostic value of the NLR measurement [19]. Our results show that the NLR–LMR index as obtained 24 h post-lysis categorized patients according to 3-months outcome more precisely and with better diagnostic accuracy.

## 5. Conclusions

In conclusion, by combining NLR and LMR results of AIS patients as obtained 24 h after thrombolysis, the estimation of patient outcomes can be significantly improved. The combination of high NLR–low LMR was found to be an independent risk factor for poor outcome at 3 months post-event. Our study showed that, owing to higher NLR and decreased LMR, a high NLR–low LMR in patients with similar stroke severity was inclined to have poor outcomes. The predicting effect of NLR–LMR ratio on stroke prognosis still needs to be confirmed in more studies in various clinical settings. 

## Figures and Tables

**Table 1:** Baseline characteristics of enrolled patients according to long term outcomes (modified Rankin Scale at 90 days post event).

	All Patients *n* = 285	Good Outcome (mRS = 0–1) *n* = 190	Poor Outcome (mRS = 2–6) *n* = 95	*p* Value
Demographic characteristics				
Age (years)	66 ± 12.9	62.8 ± 12.9	72.0 ± 10.2	<0.001
Gender, male (%)	159 (55.8)	107 (56.3)	52 (54.7)	0.802
BMI (kg/m^2^)	28.5 ± 5.9	28.5 ± 5.6	28.4 ± 6.5	0.900
Baseline laboratory results				
hsCRP (g/L)	2.8 (1.4–6.0)	2.5 (1.3–5.2)	3.5 (1.7–7.7)	0.060
White blood cell count (G/L)	8.1 (6.5–9.9)	8.04 (6.45–9.59)	8.15 (6.48–10.33)	0.455
Neutrophil count (G/L)	5.2 (4.0–7.1)	5.12 (3.99–6.86)	5.62 (4.17–7.55)	0.157
Lymphocyte count (G/L)	1.7 (1.2–2.3)	1.77 (1.31–2.3)	1.61 (1.15–2.24)	0.053
Monocyte count (G/L)	0.56 (0.44–0.69)	0.54 (0.43–0.69)	0.58 (0.47–0.71)	0.164
NLR	2.9 (1.94–4.82)	2.72 (1.86–4.66)	3.18 (2.17–5.94)	0.036
LMR	3.22 (2.42–4.29)	3.41 (2.51–4.55)	2.97 (1.87–3.92)	0.005
Vascular risk factors, *n* (%)				
Smoking				
Non-smoker	204 (71.6)	131 (68.8)	73 (76.8)	0.152
Previous smoker	2 (0.7)	2 (1.1)	0	
Current smoker	79 (27.7)	57 (30.2)	22 (23.2)	
Previous stroke/TIA	67 (23.5)	38 (20)	29 (30.5)	0.055
Atrial fibrillation	29 (10.2)	14 (7.4)	15 (15.8)	0.026
PAD	9 (3.2)	6 (3.2)	3 (3.2)	1.000
Hyperlipidemia	181 (63.5)	123 (64.7)	58 (61.0)	0.602
DM	71 (24.9)	41 (21.6)	30 (31.6)	0.081
Hypertension	246 (86.3)	158 (83.2)	88 (92.6)	0.029
Therapy at stroke onset, *n* (%)				
ACE inhibitor	148 (51.9)	92 (48.4)	56 (58.9)	0.103
Diuretic	118 (41.4)	71 (37.4)	48 (50.5)	0.056
Beta blocker	97 (34)	62 (32.6)	35 (36.8)	0.509
Calcium channel blocker	69 (24.2)	46 (24.2)	23 (24.2)	1.000
Alfa blocker	23 (8.1)	14 (7.4)	9 (9.5)	0.645
Hypertension therapy	189 (66.3)	121 (63.7)	68 (71.6)	0.231
Acetylsalicylic acid	86 (30.2)	52 (27.4)	34 (35.8)	0.171
Clopidogrel	23 (8.1)	16 (8.4)	7 (7.4)	0.822
Anticoagulant therapy, *n* (%)				
Vitamin K antagonist	9 (3.2)	5 (2.6)	4 (4.2)	
Direct thrombin inhibitor	1 (0.4)	1 (0.5)	0	
Direct factor Xa inhibitor	0	0	0	
Low molecular weight heparin	3 (1.1)	2 (1.1)	1 (1.1)	
Lipid lowering therapy, *n* (%)	78 (25)	44 (23.3)	27 (28.4)	0.384
Anti-diabetic therapy, *n* (%)	52 (17)	27 (14.2)	21 (22.1)	0.094
Stroke severity, *n* (%)				
NIHSS at day 1				
0–5	110 (38.7)	93 (48.9)	17 (18.1)	<0.001
6–10	98 (34.5)	65 (34.2)	33 (35.1)	
11–15	50 (17.6)	24 (12.6)	26 (27.7)	
>15	26 (9.2)	8 (4.2)	18 (19.1)	
NIHSS at day 7				
0–5	129 (46.9)	109 (57.7)	20 (23.3)	<0.001
6–10	113 (41.1)	77 (40.7)	36 (41.9)	
11–15	24 (8.7)	3 (1.6)	21 (24.4)	
>15	9 (3.3)	0	9 (10.5)	
Hemorrhagic transformation, *n* (%)				
aSICH	13 (4.6)	4 (2.1)	9 (9.5)	0.110
SICH	7 (2.5)	0	7 (7.4)	
Stroke localization, *n* (%)				
ICA	193 (67.7)	112 (58.9)	81 (85.3)	<0.001
VB	92 (32.3)	78 (41.1)	14 (14.7)	
Stroke etiology (TOAST), *n* (%)				
Large-artery atherosclerosis	62 (21.8)	55 (28.9)	7 (7.4)	<0.001
Small-vessel occlusion	103 (36.1)	59 (31.1)	44 (46.3)	
Cardioembolic	23 (8.1)	17 (8.9)	6 (6.3)	
Other/undetermined	97 (34)	59 (31.1)	38 (40)	

**Table 2:** Leukocyte counts and ratios in acute ischemic stroke patients before and 24 h after thrombolysis.

	Before Thrombolysis	24 h after Thrombolysis	*p* Value
Neutrophil (G/L)	5.24 (4.04–7.14)	6.26 (4.7–8.3)	<0.001
Lymphocyte (G/L)	1.74 (1.25–2.3)	1.69 (1.28–2.15)	0.061
Monocyte (G/L)	0.56 (0.44–0.69)	0.66 (0.53–0.83)	<0.001
NLR	2.9 (1.94–4.82)	3.58 (2.48–5.6)	<0.001
LMR	3.22 (2.42–4.29)	2.58 (1.74–3.56)	<0.001

NLR: neutrophil–lymphocyte ratio. LMR: lymphocyte–monocyte ratio. Statistics: Wilcoxon Signed Rank test.

**Table 3:** Leukocyte counts and ratios at admission and 24 h after thrombolysis according to stroke severity at admission and thrombolysis safety.

	Time of Blood Sampling	Neutrophil (G/L)	Lymphocyte (G/L)	Monocyte (G/L)	NLR	LMR
Hemorrhagic transformation according to ECASS II	At admission					
	No hemorrhage (*n* = 264)	5.2 (4.1–7.1)	1.7 (1.2–2.3)	0.56 (0.44–0.70)	2.88 (1.93–4.82)	3.22 (2.42–4.30)
	aSICH (*n* = 13)	5.3 (3.8–7.2)	1.7 (1.3–1.9)	0.45 (0.39–0.58)	3.07 (2.32–6.50)	3.82 (2.68–5.10)
	SICH (*n* = 7)	6.2 (3.6–8.0)	1.8 (1.4–2.2)	0.60 (0.53–0.68)	3.41 (1.96–4.54)	2.97 (2.56–3.91)
	*p* value	0.987	0.688	0.152	0.805	0.551
	24 h after thrombolysis					
	No hemorrhage (*n* = 264)	6.1 (4.6–8.2)	1.7 (1.3–2.2)	0.66 (0.52–0.83)	3.44 (2.45–5.20)	2.63 (1.75–3.59)
	aSICH (*n* = 13)	8.2 (6.6–9.1)	1.3 (1.1–1.9)	0.69 (0.62–0.87)	5.63 (3.31–8.58)	2.07 (1.2–2.59)
	SICH (*n* = 7)	9.7 (7.3–15.4)	1.3 (0.8–2.2)	0.91 (0.80–1.17)	7.12 (4.15–19.7)	1.51 (0.8–2.04)
	*p* value	0.002	0.091	0.030	0.002	0.005
Stroke severity	At admission					
	NIHSS 0–5 (*n* = 110)	5.1 (4.0–7.0)	1.8 (1.4–2.4)	0.57 (0.44–0.71)	2.75 (1.81–3.98)	3.45 (2.51–4.51)
	NIHSS 6–10 (*n* = 97)	5.5 (4.4–6.8)	1.7 (1.2–2.3)	0.57 (0.45–0.70)	2.78 (2.00–4.95)	3.01 (2.33–4.34)
	NIHSS 11–16 (*n* = 50)	5.1 (4.0–7.5)	1.6 (1.2–2.1)	0.53 (0.42–0.66)	2.99 (2.08–6.56)	3.11 (2.41–4.13)
	NIHSS > 16 (*n* = 25)	5.3 (3.6–6.9)	1.6 (1.1–1.9)	0.55 (0.44–0.63)	3.27 (2.10–5.73)	3.04 (2.36–4.06)
	*p* value	0.782	0.067	0.581	0.330	0.441
	24 h after thrombolysis					
	NIHSS 0–5 (*n* = 110)	5.4 (4.3–7.5)	1.8 (1.4–2.4)	0.61 (0.49–0.79)	3.08 (2.10–4.47)	2.95 (2.27–3.92)
	NIHSS 6–10 (*n* = 97)	6.4 (4.7–8.0)	1.7 (1.4–2.2)	0.66 (0.56–0.82)	3.30 (2.48–5.17)	2.54 (1.85–3.59)
	NIHSS 11–16 (*n* = 50)	7.7 (5.0–9.7)	1.4 (1.2–2.0)	0.67 (0.56–0.85)	4.66 (3.04–6.85)	2.26 (1.67–2.87)
	NIHSS > 16 (*n* = 25)	9.7 (7.2–13.4)	1.2 (0.9–1.7)	0.83 (0.68–1.08)	8.4 (4.05–12.98)	1.34 (1.04–1.87)
	*p* value	<0.001	<0.001	0.004	<0.001	<0.001

Data depicted as median (inter-quartile range). NLR: neutrophil–lymphocyte ratio. LMR: lymphocyte–monocyte ratio. aSICH: asymptomatic intracerebral hemorrhage. SICH: symptomatic intracerebral hemorrhage. ECASS II: European Co-operative Acute Stroke Study-II. NIHSS: National Institutes of Health Stroke Scale. Statistics: Kruskal–Wallis.

**Table 4:** Association of NLR–LMR combinations at admission and 24 h after thrombolysis with poor functional outcome (mRS 2) at 3 months post-event.

Characteristics	Univariate Analysis, Crude OR (95% CI)	*p* Value	Multivariate Analysis, Adjusted OR (95% CI) ^a^	*p* Value
At admission				
Low NLR–High LMR (*n* = 211)	Ref	-	Ref	-
High NLR–High LMR (*n* = 22)	0.766 (0.310–1.891)	*p* = 0.563	0.338 (0.075–1.530)	*p* = 0.159
Low NLR–Low LMR (*n* = 19)	0.993 (0.516–1.914)	*p* = 0.507	1.486 (0.462–4.779)	*p* = 0.507
High NLR–Low LMR (*n* = 33)	5.496 (3.236–9.336)	*p* < 0.001	3.049 (1.205–7.714)	*p* = 0.019
On day 1				
Low NLR–High LMR (*n* = 178)	Ref	-	Ref	-
High NLR–High LMR (*n* = 10)	1.412 (0.555–3.591)	*p* = 0.469	4.860 (0.816–28.944)	*p* = 0.082
Low NLR–Low LMR (*n* = 35)	1.831 (0.914–3.671)	*p* = 0.088	1.168 (0.439–3.107)	*p* = 0.755
High NLR–Low LMR (*n* = 62)	10.13 (5.685–18.066)	*p* < 0.001	6.353 (2.774–14.548)	*p* < 0.001

CI: confidence interval. OR: odds ratio. NLR: neutrophil–lymphocyte ratio. LMR: monocyte–lymphocyte ratio. ^a^ Controlled for: age, atrial fibrillation, hypertension, NIHSS, hemorrhagic transformation, and stroke localization.

## Data Availability

The data presented in this study are available upon request from the corresponding author according to “MDPI Research Data Policies” at https://www.mdpi.com/ethics. Accessed on 1 July 2022.

## Supplementary Materials

The following supporting information can be downloaded at: https://www.mdpi.com/article/10.3390/jpm12081221/s1, Table S1: Leukocyte counts and ratios at admission according to stroke etiology; Table S2: Leukocyte counts and ratios according to ASPECTS at admission and 24 h after thrombolysis; Table S3: Univariable and multivariable logistic regression analyses depicting the associations of admission NLR, LMR, and other baseline characteristics with functional dependence (mRS ≥ 2) at 3 months post-event; Table S4: Univariable and multivariable logistic regression analyses depicting the associations of day 1 NLR, LMR, and baseline characteristics with functional independence at 3 months post-event (mRS ≥ 2).
