# Supplementary material for: High Neutrophil–Lymphocyte Ratio and Low Lymphocyte–Monocyte Ratio Combination after Thrombolysis Is a Potential Predictor of Poor Functional Outcome of Acute Ischemic Stroke
Source: J Pers Med. 2022 Jul 27;12(8):1221. doi: 10.3390/jpm12081221 (PMC9332832; doi:10.3390/jpm12081221)
Supplement: Supplementary file 1 [file jpm-12-01221-s001.zip › jpm-1802436-supplementary.pdf]

Table S1. Leukocyte counts and ratios at admission according to stroke etiology.

Stroke etiology (TOAST)	Neutrophil (G/L)	Lymphocyte (G/L)	Monocyte (G/L)	NLR	LMR
Large-artery atherosclerosis (n = 62)	4.9 (4.1-6.7)	1.8 (1.5-2.3)	0.57 (0.42-0.70)	2.68 (1.81-3.56)	3.56 (2.79-4.34)
Small-vessel occlusion (n = 103)	5.4 (3.8-7.4)	1.7 (1.2-2.3)	0.53 (0.43-0.71)	3.00 (1.95-5.03)	3.20 (2.31-4.42)
Cardioembolic (n = 23)	4.9 (3.9-6.7)	1.7 (1.3-1.9)	0.49 (0.40-0.65)	2.89 (2.10-4.26)	2.97 (2.63-4.24)
Other/undetermined (n = 97)	5.4 (4.2-7.2)	1.7 (1.2-2.3)	0.58 (0.47-0.68)	3.12 (1.94-5.52)	3.05 (2.36-4.04)
p value	0.623	0.392	0.635	0.392	0.409

Data are depicted as median (interquartile range), NLR: neutrophil- lymphocyte ratio, LMR: lymphocyte- monocyte ratio, TOAST: Trial of ORG 10172 in Acute Stroke; Statistics: Kruskal-Wallis.

Table S2. Leukocyte counts and ratios according to ASPECTS at admission and 24h after thrombolysis.

	Groups	Neutrophil (G/L)	Lymphocyte (G/)	Monocyte (G/L)	NLR	LMR
On admission	ASPECTS 10-8 (n = 5)	5.3 (4.1-6.9)	1.8 (1.3-2.3)	0.56 (0.45-0.69)	2.87 (1.83-4.95)	3.23 (2.41-4.23)
	ASPECTS 7-0 (n = 151)	6.3 (6.2-6.4)	1.6 (1.3-1.8)	0.64 (0.58-0.69)	3.41 (3.18-4.78)	2.56 (1.9-2.78)
p Value		0.193	0.740	0.270	0.271	0.298
At day 1	ASPECTS 10-8 (n = 38)	6.9 (4.9-8.3)	1.6 (1.3-2.1)	0.68 (0.53-0.89)	3.8 (2.55-6.04)	2.56 (1.62-3.47)
	ASPECTS 7-0 (n = 116)	8.5 (6.4-10.3)	1.5 (1.0-1.9)	0.74 (0.61-0.92)	5.59 (3.13-9.26)	2.01 (1.26-2.94)
p Value		0.002	0.132	0.218	0.004	0.072

Data depicted as median (inter-quartile range). NLR: neutrophil-lymphocyte ratio, LMR: lymphocyte-monocyte ratio. Statistics: Mann-Whitney U test.

Table S3. Univariable and multivariable logistic regression analyses depicting the associations of admission NLR, LMR and other baseline characteristics with functional dependence (mRS ≥ 2) at 3 months post-event.

**Parameters**	**Univariable Logistic Regression Analysis**		**Multivariable Logistic Regression Analysis**	
	**OR (95%CI)**	***p* value**	**OR (95%CI)**	***p* value**
Age (year)	1.076 (1.048-1.105)	0.001	1.052 (1.010-1-096)	0.015
NLR	1.046 (0.988-1.108)	0.121	1.044 (0.894-1.219)	0.584
LMR	0.755 (0.631-0.903)	0.002	0.534 (0.244-1.166)	0.115
Atrial fibrillation *n* (%)	1.656 (1.086-2.527)	0.019	0.503 (0.233-1.085)	0.080
Hypertension *n* (%)	2.546 (1.079-6.007)	0.033	0.695 (0.168-2.879)	0.616
NIHSS at day 1	2.333 (1.757-3.098)	< 0.001	2.448 (1.537-3.898)	< 0.001
NIHSS at day 7	4.613 (2.949-7.215)	< 0.001	8.762 (4.346-17.668)	< 0.001
Hemorrhagic transformation	6.874 (2.441-19.357)	< 0.001	6.608 (1.334-32.747)	0.021
Stroke localization	0.248 (0.131-0.469)	< 0.001	0.333 (0.122-0.909)	0.032

LMR: lymphocyte-monocyte ratio. mRS: modified Rankin Scale. NIHSS: National Institutes of Health Stroke Scale. NLR: neutrophil-lymphocyte ratio.

Table S4. Univariable and multivariable logistic regression analyses depicting the associations of day 1 NLR, LMR and baseline characteristics with functional independence at 3 months post-event (mRS ≥ 2).

**Parameters**	**Univariable Logistic Regression Analysis**		**Multivariable Logistic Regression Analysis**	
	**OR (95%CI)**	***p* Value**	**OR (95%CI)**	***p* Value**
Age (year)	1.076 (1.048-1.105)	< 0.001	1.056 (1.011-1.111)	0.014
hsCRP (g/L)	1.011 (1.001-1.022)	0.038	1.005 (0.979-1.031)	0.722
NLR	1.417 (1.266-1.585)	< 0.001	1.416 (0.963-2.083)	0.077
LMR	0.453 (0.347-0.591)	< 0.001	0.311 (0.103-1.413)	0.056
Atrial fibrillation. No. (%)	1.656 (1.086-2.527)	0.019	0.347 (0.191-0.981)	0.033
Hypertension. No. (%)	2.546 (1.079-6.007)	0.033	0.541 (0.117-2.493)	0.430
NIHSS at day 1	2.333 (1.757-3.098)	< 0.001	0.925 (1.420-3.948)	0.177
NIHSS at day 7	4.613 (2.949-7.215)	< 0.001	1.537 (1.335-1.769)	< 0.001
Hemorrhagic transformation	6.874 (2.441-19.357)	< 0.001	4.102 (0.589-28.579)	0.154
Stroke localization	0.248 (0.131-0.469)	< 0.001	0.393 (0.137-1.131)	0.083

LMR: lymphocyte-monocyte ratio. mRS: modified Rankin Scale. NIHSS: National Institutes of Health Stroke Scale. NLR: neutrophil-lymphocyte ratio.
